# The impact of weight self-stigma on appearance anxiety among female college students: a variable-centered and person-centered analysis

**DOI:** 10.3389/fpubh.2025.1709480

**Published:** 2025-12-04

**Authors:** Luyao Xiang, Hao Gou, Chang Hu

**Affiliations:** 1 Zhuhai Campus Zunyi Medical University, Zhuhai, China; 2 College of Physical Education, Qiannan Normal University for Nationalities, Duyun, China; 3 College of Physical Education, Jiangxi Normal University, Nanchang, China

**Keywords:** weight self-stigma, appearance anxiety, body image, latent profile analysis, female college students

## Abstract

**Background:**

Appearance anxiety and weight self-stigma have become increasingly prominent issues among female university students. However, existing studies have primarily concentrated on direct associations at the variable level, with limited exploration of heterogeneous subgroups from a person-centered perspective. This study aimed to examine the mediating role of body image in the relationship between weight self-stigma and appearance anxiety and to identify potential latent categories of weight self-stigma and body image, thereby providing scientific guidance for the prevention and improvement of appearance anxiety in female university students.

**Methods:**

From November 2024 to March 2025, a purposive sampling strategy was employed. A total of 1,368 female university students were recruited from 12 comprehensive universities located in six provinces of central and southern China (Hunan, Guizhou, Yunnan, Guangdong, Jiangxi, and Jiangsu). Participants completed the appearance anxiety scale, the weight self-stigma questionnaire, and the body image scale.

**Results:**

(1) Weight self-stigma positively predicted appearance anxiety among female university students, with body image playing a partial mediating role (*β* = 0.250, *p <* 0.001); (2) Three latent patterns of weight self-stigma and body image were identified: low stigma—high body image (23.4%), moderate stigma-moderate body image (27.7%), and high stigma—low body image (48.9%); (3) Compared with the “low stigma—high body image” group, the other two categories significantly and positively predicted appearance anxiety (*p <* 0.001).

**Conclusion:**

Weight self-stigma and body image among female university students demonstrate notable heterogeneity, and body image serves as a mediator in the relationship between weight self-stigma and appearance anxiety.

## 1 Introduction

In recent years, in the era of social media–dominated visual culture, women’s bodies have increasingly become objects of gaze, evaluation, and regulation (1, 2). Within East Asian societies, the aesthetic ideology of “thinness as beauty” transforms weight management into a symbol of moral self-discipline (3), thereby trapping women in the cognitive pitfall of “body self-objectification” (4). As heavy users of social media and individuals navigating a transitional stage of identity (5), female college students experience weight self-stigma not only as a fear of being labeled overweight but also as an internalized self-denigration of “non-normative body shapes (6),” which subsequently induces persistent appearance anxiety (7). This phenomenon is particularly pronounced in the Chinese context, where the prevailing ideal of “fair, slim, and youthful” beauty is deeply ingrained (8), yet it has received insufficient scholarly attention.

Aesthetic ideals such as the “palm-sized face,” “A4 waist,” and “cartoon-like legs,” which reduce body parts to measurable parameters or symbolic standards (9), are continuously produced, disseminated, and reinforced through social media, rendering appearance anxiety a typical feature of modern psychological distress (10, 11). Appearance anxiety refers to the dual psychological stress experienced during the process of aesthetic socialization: it encompasses both appearance evaluation anxiety triggered by social scrutiny and self-evaluation anxiety arising from the discrepancy between internalized aesthetic standards and one’s actual appearance (12). As a psychosocial phenomenon deeply embedded in cultural contexts, appearance anxiety is particularly prominent among young women who are strongly influenced by prevailing beauty norms (13, 14). Unlike clinically diagnosed anxiety disorders, appearance anxiety reflects the state of worry, tension, and unease stemming from aesthetic pressures within specific sociocultural environments (15). Empirical studies reveal that high levels of appearance anxiety are prevalent among contemporary youth and are closely associated with a range of adverse psychosocial outcomes, including reduced self-esteem (16), increased social inhibition (17), and intensified tendencies toward social withdrawal (18). Although Appearance anxiety may temporarily heighten motivation for appearance enhancement, research demonstrates that it ultimately produces multidimensional impairments in mental and physical functioning, with individuals experiencing elevated appearance anxiety often exhibiting lower self-esteem (19), stronger social inhibition (20), and more pronounced social withdrawal tendencies (19). Among female college students, the combined influences of social media and mainstream cultural discourses continually amplify attention to physical appearance (21). Coupled with algorithm-driven precision targeting in the era of big data, these dynamics invisibly aggravate their levels of Appearance Anxiety, making this issue an urgent and pressing concern.

### 1.1 Weight self-stigma and appearance anxiety

Weight self-stigma refers to the process by which individuals with different body weight statuses internalize others’ negative weight-related evaluations, resulting in self-devaluation (22). Under the aesthetic hegemony of “thinness as justice,” weight self-stigma has gone beyond mere body dissatisfaction and become, as Foucault described, a “technology of self-discipline” (23). Specifically, female college students exhibit an obsessive pursuit of a subjectively constructed “standardized body shape” that is often more stringent than health-based norms (24). Such self-imposed demands, driven by internalized stigma, create a state of cognitive dissonance when juxtaposed with the clinical evidence showing that the majority of individuals’ BMIs fall within a normal and healthy range (25). To alleviate the discomfort caused by this dissonance, they tend to focus more on their physical appearance and attempt to compensate for perceived “deficiencies” in weight through other means, thereby intensifying appearance anxiety (26). According to the self-objectification theory (27), individuals, especially women, tend to ground their self-worth in others’ evaluations of their appearance, viewing themselves as objects of observation rather than autonomous subjects. This self-objectification heightens preoccupation with body image, leading to anxiety, decision-making difficulties, and emotional exhaustion (28). Examples include excessive modification of selfies or avoiding social interactions due to fear of negative evaluation, both of which reflect an overreliance on external gazes (29). Empirical research further supports the link between weight-related factors and Appearance Anxiety. For instance, Göbel et al. (30) found that BMI mediates the relationship between women’s social appearance anxiety, defined as anxiety during social interactions stemming from concerns about negative evaluations of appearance and self-esteem (31). This finding suggests that weight-related factors may influence individuals’ specific anxiety experiences in social contexts, which in turn connect to broader aspects of self-esteem, providing indirect evidence for the association between weight-related psychology and appearance anxiety (32). Therefore, this study proposes Hypothesis 1 (H1): Weight self-stigma is positively associated with appearance anxiety among female college students.

### 1.2 Body image, weight self-stigma, and appearance anxiety

Body image refers to the comprehensive manifestation of an individual’s cognition, emotions, and attitudes toward their body, encompassing perceptions and evaluations of physical appearance, functionality, and social meaning. It exerts broad influences on self-identity, mental health, and behavioral lifestyle (33, 34). Social Comparison Theory suggests that when individuals internalize prevailing societal beauty standards and engage in upward comparisons with others, they are prone to cognitive dissonance, which leads to body dissatisfaction (35, 36). Meanwhile, the cognitive-behavioral model explains that negative body schemata magnify perceived appearance flaws, thereby triggering appearance anxiety (25, 37). Empirical research has confirmed a robust association between body image dissatisfaction and elevated levels of appearance anxiety among both adolescents and adults (38).

Within the domain of weight self-stigma, sociocultural theory emphasizes that cultural prescriptions, such as the ideology of “thinness as beauty,” foster negative social stigma surrounding weight (39). Through internalization, this stigma leads to self-objectification, in which individuals begin to regard their bodies as objects for others’ observation and evaluation. Such a self-objectifying perspective distorts one’s perception and evaluation of the body (15), undermines positive body image, and heightens preoccupation with perceived appearance flaws, directly contributing to increased appearance anxiety (40). Self-discrepancy theory further elucidates that weight self-stigma often co-occurs with cognitive biases, whereby individuals attribute weight-related concerns or failure to achieve an ideal body shape to personal moral shortcomings (41). This moralization of weight engenders intense feelings of shame and self-criticism, thereby diminishing satisfaction with Body Image (42, 43). Persistent dissatisfaction and negative evaluations of the body constitute core experiential components of appearance anxiety (44). Research demonstrates that improving body image effectively alleviates weight self-stigma, underscoring its mediating function within the pathological mechanism (45). For example, one study found that individuals with higher body mass index (BMI) often exhibit negative body image and weight self-stigma (46). Moreover, satisfaction with body image is negatively associated with weight self-stigma, and interventions targeting body image improvement can mitigate weight self-stigma (47). Therefore, this study proposes Hypothesis 2 (H2): body image mediates the relationship between weight self-stigma and appearance anxiety among female college students.

### 1.3 A person-centered perspective

The above discussion primarily adopts a variable-centered perspective to examine the mechanisms through which weight self-stigma influences appearance anxiety. However, variable-centered findings are largely derived from the average level across the entire sample, and such “averages” do not adequately capture individual heterogeneity (48). Therefore, exploring the relationship between weight self-stigma and appearance anxiety among female college students from both variable-centered and person-centered perspectives not only elucidates the systematic impact of sociocultural pressures on women’s psychological experiences but also identifies the heterogeneous characteristics of potential subgroups (49). This dual approach provides stratified intervention pathways to disrupt the vicious cycle of “stigma internalization-anxiety escalation,” thereby contributing to the construction of explanatory models and coping strategies grounded in the Chinese sociocultural context.

### 1.4 Exploring the relationship between weight self-stigma and appearance anxiety: a mixed-methods approach

This study distinguishes itself by using both variable-centered and person-centered approaches. While previous research has focused on variable-level relationships, our study employs latent profile analysis to explore the heterogeneity of weight self-stigma and body image among female university students (50). This method helps us better understand the impact of weight self-stigma on appearance anxiety and identify distinct subgroups within this population (51). However, it is important to acknowledge that the homogeneity of the sample, consisting exclusively of female students from comprehensive universities in central and southern China, creates a narrow demographic scope, which may limit the generalizability of our findings to broader populations. The main research question is, “How does weight self-stigma influence appearance anxiety among female university students, and what role does body image play in this relationship?” To refine this, we pose the following subquestions: (1) What are the latent profiles of weight self-stigma and body image among female students? (2) How do these profiles predict appearance anxiety?

## 2 Methods

### 2.1 Participant

A purposive sampling approach was chosen to ensure participants were selected based on their relevance to the research question, as female university students are particularly affected by body image concerns under societal pressures. The purposive sampling strategy enabled us to focus on a group directly aligned with the study’s objectives, enhancing the relevance of the findings. A survey was conducted from November 2024 to March 2025 among female university students in six provinces of central and southern China (Hunan, Guizhou, Yunnan, Guangdong, Jiangxi, and Jiangsu). In each province, two comprehensive universities were selected: one located in a provincial capital, sub-provincial city, or an economically developed urban area, and the other in a non-capital or local city. This design ensured regional diversity and indirectly captured different socio-geographic backgrounds.

Before the formal survey, investigators received standardized training and conducted a pilot study with a small sample to confirm that the Cronbach’s *α* coefficients of the scales exceeded 0.70. Only after this validation was the large-scale survey carried out. Before completing the questionnaire, participants were informed about the study’s purpose, significance, and procedures and provided informed consent. The survey was supervised jointly by class counselors and trained research assistants. Upon completion, all questionnaires were checked, verified, and cross-examined; incomplete or missing items were supplemented or excluded to ensure data integrity and reliability. The questionnaires were distributed via the online platform Wenjuanxing, promoted through student unions, counselors, and social media groups. A total of 1,500 questionnaires were distributed, and 1,426 valid responses were returned (response rate = 95%). After excluding patterned responses and questionnaires with more than 10% missing items, 1,368 valid samples were retained for analysis (validity rate = 96%). Demographic characteristics of the participants were as follows: the mean age was 19.81 years (SD = 1.62), with 37.6% sophomores, 28.3% freshmen, 23.3% juniors, and 10.8% seniors. Regarding household registration, 64.1% of participants were from rural areas, while 35.9% were from urban areas (see Table 1).

**Table 1:** Participant demographics.

Characteristic	Mean (SD) or *n* (%)
Age	19.81 (1.62)
Grade	
Freshman	387 (28.3%)
Sophomore	515 (37.6%)
Junior	319 (23.3%)
Senior	147 (10.8%)
Household registration	
Urban	491 (35.9%)
Rural	877 (64.1%)

### 2.2 Measures

#### 2.2.1 Weight self-stigma scale

In this study, the weight self-stigma scale, developed by Lillis et al. (52) was adopted. The scale consists of two core dimensions: self-devaluation and fear of enacted stigma. It includes 12 items rated on a 5-point Likert scale, ranging from 1 (strongly disagree) to 5 (strongly agree). Scores from all items were summed to generate a total score, with higher scores indicating greater weight self-stigma. To ensure the scale’s relevance and applicability to the Chinese population, a translation and cultural adaptation process was conducted. The scale was translated from English to Chinese using a forward-backward translation method. First, two bilingual researchers independently translated the scale into Chinese, and a third bilingual researcher reviewed the translations for consistency. A back-translation was conducted by two other bilingual researchers to verify the translation’s accuracy. Cultural adjustments were made as needed to ensure the content was culturally appropriate for the target population.

After localization and validation, this scale has demonstrated good reliability and validity among Chinese adolescents (53). The psychometric evaluation showed that the scale had strong internal consistency, with a Cronbach’s *α* coefficient of 0.866. Furthermore, confirmatory factor analysis (CFA) indicated satisfactory model fit, with indices meeting accepted standards: χ^2^/df = 1.632, RMSEA = 0.021, SRMR = 0.017, CFI = 0.994, and TLI = 0.993. The Self-Devaluation dimension includes items 1–6, with a Cronbach’s *α* of 0.844, and the Fear of Enacted Stigma dimension includes items 7–12, with a Cronbach’s α of 0.846. These results confirm the reliability and validity of the scale for use in the current study.

#### 2.2.2 Body image scale

The body image scale (54), developed and later cross-culturally adapted, was employed in this study (55). The instrument consists of six items rated on a 9-point Likert scale, ranging from 1 (very dissatisfied) to 9 (very satisfied), with higher scores indicating greater acceptance of one’s body image. The scale is unidimensional, assessing overall body image satisfaction. The Chinese version of the Body Image Scale was developed through a rigorous translation and adaptation process. Like the other scales, it was translated and culturally adapted using a forward-backward translation method to ensure its relevance and applicability to the Chinese context. The scale was first translated from English to Chinese by bilingual researchers, and a back-translation was conducted to verify the accuracy of the translation. Adjustments were made to ensure the scale’s cultural relevance and clarity for the Chinese population. Psychometric evaluation of the Chinese version showed strong internal consistency (Cronbach’s *α* = 0.914). It confirmed a satisfactory model fit through confirmatory factor analysis (CFA), with the following indices: χ^2^/df = 3.626, RMSEA = 0.044, SRMR = 0.010, CFI = 0.995, and TLI = 0.992. These results indicate that the body image scale is reliable and valid for use with Chinese female university students.

#### 2.2.3 Appearance anxiety scale

This study employed the short version of the appearance anxiety scale, which was culturally adapted and revised (56). The scale is designed to measure individuals’ tendencies to experience worry, tension, and uneasiness about their appearance under sociocultural aesthetic pressures, rather than to diagnose clinical anxiety disorders. It consists of 14 items rated on a 5-point Likert scale, ranging from 1 (never) to 5 (always). Higher total scores indicate stronger experiences of appearance-related anxiety. The scale is unidimensional, assessing overall appearance anxiety. Like the other scales, the appearance anxiety scale was translated and culturally adapted using a forward-backward translation method to ensure its relevance to the Chinese context. The scale’s psychometric properties were assessed, showing good reliability (Cronbach’s *α* = 0.907) and satisfactory CFA fit indices: χ^2^/df = 1.505, RMSEA = 0.019, SRMR = 0.016, CFI = 0.994, and TLI = 0.993. These results confirm the scale’s robustness in measuring appearance anxiety among Chinese university students.

### 2.3 Data analysis

Descriptive statistics, correlation analyses, and multiple logistic regression analyses were conducted using SPSS 27.0. Mediation analysis was performed using the SPSS 27.0 macro PROCESS (Model 4). Confirmatory factor analysis and latent profile analysis were conducted using Mplus 8.3. Model fit was evaluated through multiple indices, including AIC, BIC, adjusted BIC (aBIC), entropy, the Lo–Mendell–Rubin likelihood ratio test (LMRT), and the bootstrap likelihood ratio test (BLRT). Lower values of AIC, BIC, and aBIC indicated better model fit, while entropy values closer to 1 reflected higher classification accuracy. Significant *p*-values (< 0.05) for LMRT and BLRT suggested that the K-class model outperformed the K–1 class model. To avoid unnecessary model segmentation, a minimum class probability of 5% was set (57).

## 3 Results

### 3.1 Common method Bias test

Harman’s single-factor analysis was conducted to examine potential common-method bias across all questionnaire items. The results indicated that four factors had eigenvalues greater than 1. The first factor accounted for 30.36% of the variance, which was below the critical threshold of 40%, indicating that common method bias was not a serious concern in this study.

### 3.2 Correlation analysis among variables

Correlation analyses were conducted on the mean scores of weight self-stigma, body image, and appearance anxiety. The results indicated that weight self-stigma was positively correlated with appearance anxiety, while body image was negatively correlated with both weight self-stigma and appearance anxiety (see Table 2).

**Table 2:** Means, standard deviations, and correlation coefficients of the variables.

Variables	M ± SD	Weight self-stigma	Body image	Appearance anxiety
Weight Self-Stigma	3.00 ± 0.88	1		
Body Image	4.97 ± 2.13	−0.453***	1	
Appearance Anxiety	3.08 ± 0.92	0.396***	−0.459***	1

*p < 0.05, **p < 0.01, ***p < 0.001. The same notation applies below.

### 3.3 Mediation model of body image in the relationship between weight self-stigma and appearance anxiety: a variable-centered analysis

**Figure 1:**
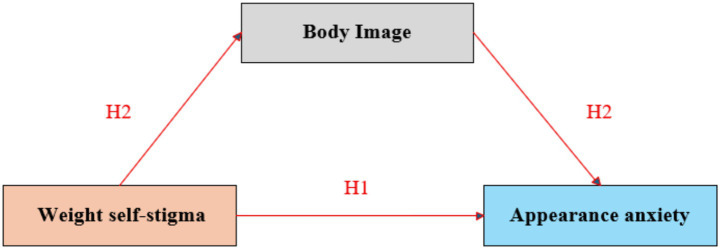
Hypothetical model.

**Figure 2:**
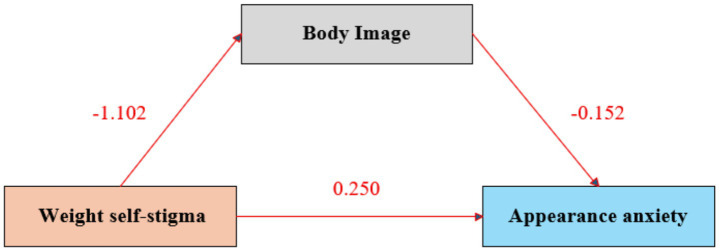
Mediation model.

Using SPSS 27.0 and the PROCESS macro (version 4.1, Model 4), the mediating effect of body image was tested with weight self-stigma as the independent variable, appearance anxiety as the dependent variable, and body image as the mediator. Table 3 and Figure 1 show that weight self-stigma significantly predicted body image (*β* = −1.102, *p <* 0.001) and appearance anxiety (*β* = 0.250, *p <* 0.001). Body image also significantly predicted appearance anxiety (*β* = −0.152, *p <* 0.001). The total effect was significant (*β* = 0.417, *p <* 0.001), with a significant indirect effect of body image (0.167, 95% CI: 0.136–0.203). Model fit indices indicated good explanatory power (R^2^ for the mediator model = 0.205, R^2^ for the outcome model = 0.255, *F* = 352.160, *p <* 0.001). These findings emphasize the importance of addressing body image in reducing appearance anxiety (Figure 2).

**Table 3:** Mediating effect of body image.

Model paths	Fit indices				Significance of coefficients			
	R	R^2^	*F*	*p*	*β*	(95%CI)		*p*
Weight Self-Stigma → Body Image	0.453	0.205	352.160	<0.001	−1.102	−1.218	−0.987	<0.001
Weight Self-Stigma → Appearance Anxiety	0.505	0.255	233.920	<0.001	0.250	0.195	0.304	<0.001
Body Image → Appearance Anxiety					−0.152	−0.175	−0.130	<0.001
Weight Self-Stigma → Body Image → Appearance Anxiety	0.396	0.157	254.102	<0.001	Total Effect:0.417	0.366	0.469	<0.001
					Indirect Effect:0.167	0.136	0.203	<0.001

### 3.4 Associative effects of latent classes of weight self-stigma and body image on appearance anxiety: a person-centered analysis

In this study, item scores of weight self-stigma and body image were used as observed indicators to construct latent profile models ranging from one to five classes. The results of latent profile analysis showed that as the number of profiles increased, the AIC, BIC, and aBIC values consistently decreased. Among all models, the three-profile solution yielded the highest entropy value, and both the LMR test (*p <* 0.001) and BLRT (*p <* 0.001) were statistically significant. Based on a comprehensive consideration of these fit indices, the three-class profile model was determined to be the most appropriate solution (Table 4).

**Table 4:** Fit indices of latent profile analysis for weight self-stigma and body image.

Class	AIC	BIC	aBIC	Entropy	LMRP	BLPTP	Class probability (%)
1	95804.450	95992.410	95878.052	/	/	/	1.000
2	90711.373	90998.534	90823.821	0.898	<0.001	<0.001	67.325/32.675
3	88628.834	89015.196	88780.128	0.900	<0.001	<0.001	23.392/27.705/48.904
4	87266.746	87752.309	87456.886	0.894	<0.001	<0.001	22.003/34.649/21.564/21.784
5	86547.006	87131.770	86775.991	0.882	0.112	<0.001	21.637/16.886/23.684/20.833/16.959

**Figure 3:**
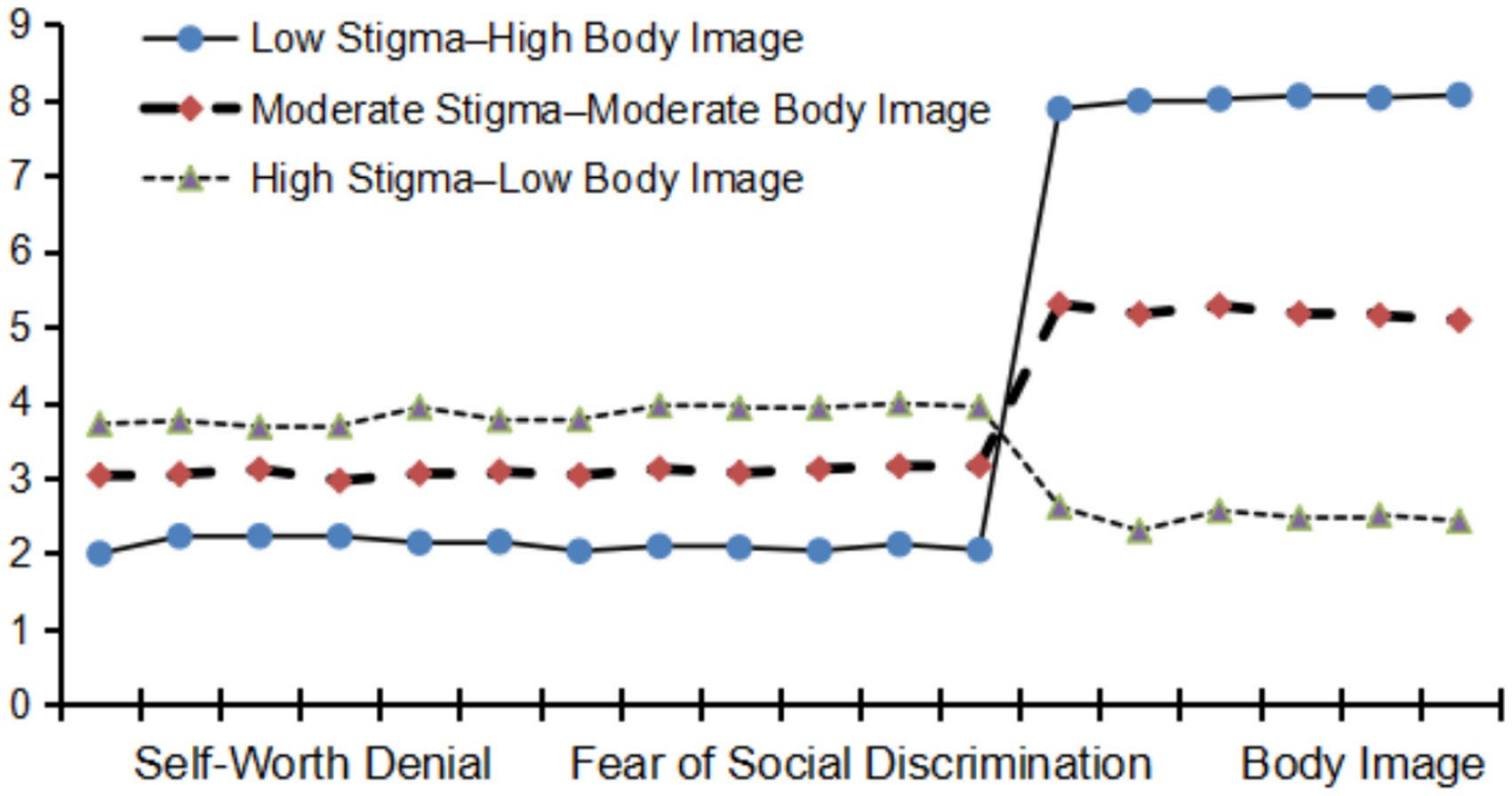
Latent profile analysis of three classes of weight self-stigma and body image among female university students.

Based on weight self-stigma and body image, three latent classes were identified and labeled accordingly (see Figure 3). The C1 group included 320 female university students (23.4% of the total sample). Their scores on the two dimensions of weight self-stigma ranged from 1.994 to 2.225 (low level), while body image scores ranged from 7.884 to 8.066 (high level). This group was therefore labeled as *Low Stigma–High Body Image*. The C2 group comprised 379 female university students (27.7%). Their scores on weight self-stigma (2.958–3.158) and body image (5.083–5.294) were both at a moderate level, indicating a *Moderate Stigma–Moderate Body Image*. The C3 group included 669 female university students (48.9%). Their scores on the two dimensions of weight self-stigma ranged from 3.672 to 3.954 (high level), while body image scores ranged from 2.297 to 2.614 (low level). Accordingly, this group was labeled as *High Stigma–Low Body Image.*

To further examine the relationship between the three latent classes and appearance anxiety, multinomial logistic regression was performed with the three categories as dependent variables, using the Low *Stigma–High Body Imag*e group as the reference category. The results indicated that different profiles of weight self-stigma and body image were significantly associated with appearance anxiety (*p <* 0.001) (Table 5).

**Table 5:** Multinomial logistic regression results of weight self-stigma and body image latent profiles.

Predictor variables	Moderate stigma–moderate body image					High stigma–low body image				
	B	*p*	OR	95% CI		B	*p*	OR	95% CI	
				Lower bound	Upper bound				Lower bound	Upper bound
Appearance anxiety	0.651	<0.001	1.917	1.640	2.241	1.910	<0.001	6.750	5.333	8.544

## 4 Discussion

### 4.1 Associative effect of weight self-stigma on appearance anxiety among female university students

The results of this study demonstrated that weight self-stigma is positively associated with appearance anxiety among female university students, supporting Hypothesis H1. This finding is consistent with previous studies (58). The phenomenon can be explained from both sociocultural and psychological perspectives. First, female university students are in a developmental stage where self-identity is particularly sensitive (59). Under the combined influence of the Eastern “culture of body shame” and the “visual consumerism” of social media, they are prone to interpret weight gain or loss of weight control as a devaluation of self-worth (60). The “culture of body shame” refers to a sociocultural belief system that links deviation from mainstream body ideals with moral flaws (e.g., laziness, lack of self-discipline), thereby assigning shame to such body features (61). Meanwhile, “visual consumerism” on social media platforms transforms body image into a commodified and spectacularized symbol, serving as a key means to attract attention and stimulate consumption; in this context, users not only consume content but may themselves become “visual commodities” (62). Second, during the transitional stage from “student” to “member of society,” this population tends to place excessive emphasis on body image management (63). Through the process of weight stigma internalization, social scrutiny is transformed into self-discipline, thereby triggering persistent appearance anxiety (64).

Finally, self-objectification theory further clarifies this pathway. Weight self-stigma reflects a cognitive alienation process in which individuals reduce themselves to “objects of evaluation (27).” Female university students with appearance anxiety often engage in reflexive gaze—the internalization of societal scrutiny of the body and the continuous monitoring and evaluating of their own physical state through this external perspective (65). In doing so, they simplify the body into quantifiable indicators such as weight and waist circumference (66). As a result, cognitive resources are diverted from self-agency (e.g., academic performance, social competence) toward objectified body-related concerns (67). This mechanism of self-objectification amplifies the intensity of appearance anxiety, and through the cycle of “body shame–behavioral inhibition” (e.g., avoiding social interaction, minimizing body exposure, restricting activities due to body shame), negative attention and shame are reinforced (68). This cycle solidifies the persistence of anxiety.

### 4.2 The mediating role of body image

The mediation analysis revealed that body image mediated the relationship between weight self-stigma and appearance anxiety among female university students, supporting Hypothesis H2. This phenomenon can be explained from two theoretical perspectives. Conversely, social comparison theory posits that individuals construct self-perceptions through comparison with others (35). Female students with high weight self-stigma are more likely to engage in unfavorable comparisons with the “ideal body shape” portrayed on social media—for example, excessive focus on digitally edited body images of online influencers and the internalization of specific features such as “waist size” or “leg shape” as self-evaluation standards (69). This form of visual discipline reinforces the biased cognition of “I do not meet the standard,” thereby leading to a persistently negative body image. A longitudinal study has shown that frequent exposure to such content significantly reduces women’s body satisfaction, confirming that social comparison constitutes a core pathway through which stigma influences body image (70).

On the other hand, the self-discrepancy theory emphasizes that a gap between the “actual self” and the “ideal self” induces anxiety (41). For individuals with weight self-stigma, “thinness” is often equated with values such as “self-discipline” and “success,” while weight fluctuations are perceived as a deviation from the ideal identity (71). For example, even individuals with a normal BMI may feel self-blame due to slight weight increases (72). Such self-criticism further undermines body acceptance, forming a vicious cycle of “anxiety–control–greater anxiety.” Research indicates that the correlation between body image and anxiety among stigmatized individuals is much stronger than the correlation between anxiety and physiological indicators such as BMI (36, 73), underscoring the dominant role of cognitive mechanisms. In sum, weight stigma distorts the body reference system through social comparison and reinforces the sense of discrepancy between the actual and ideal self, ultimately catalyzing appearance anxiety (74).

### 4.3 The associative impact of different latent classes of weight self-stigma and body image on appearance anxiety

This study identified three distinct patterns of association between weight self-stigma and body image: Low Stigma–High Body Image, Moderate Stigma–Moderate Body Image, and High Stigma–Low Body Image. The distribution of these classes differed from that reported in previous research (75). One possible explanation for this discrepancy lies in the fact that prior studies have primarily focused on the linear relationships between weight stigma and negative psychological indicators such as anxiety and depression (76). By introducing body image as a positive variable, the present study uncovered an asymmetry between weight stigma and body-related cognition—namely, some individuals, despite experiencing weight stigma, may still maintain a relatively positive body image through social support, body functionality recognition, or the adoption of diversified aesthetic standards (77). For instance, certain female university students, though subject to social pressure regarding weight, may redefine the value of their bodies through activities such as dance or fitness, resulting in a paradoxical state of “stigma exists but body image remains high (78).”

Cultural background differences may also contribute to this heterogeneity. Aesthetic standards in Eastern societies are diverse and complex, encompassing not only the widely propagated “slim, fair, and young” ideal but also the pursuit of “proportionate beauty” and “healthy beauty” (79). This multiplicity of standards means that individuals facing weight self-stigma may be influenced by different aesthetic frameworks (80). On the one hand, the “slim, fair, and young” ideal, prevalent in social media and contemporary culture, intensifies weight self-stigma (81). On the other hand, traditional Eastern cultural values that emphasize proportion and health offer individuals a more inclusive aesthetic perspective (82). Thus, while the “slim, fair, and young” ideal may reinforce weight self-stigma, the notions of “proportionate beauty” and “healthy beauty” can, to some extent, mitigate its negative effects.

The results of the logistic regression analysis showed that, using the Low Stigma–High Body Image group as the reference, female students in the High Stigma–Low Body Image group exhibited significantly higher appearance anxiety. Specifically, for every one-unit increase in appearance anxiety, the probability of belonging to the High Stigma–Low Body Image group was 6.75 times that of the reference group (95% CI: 5.333–8.544). This finding is consistent with the results of the variable-centered analysis and further validates the pathway of “weight stigma → impaired body image → heightened anxiety.” Social comparison theory helps explain the stigma activation stage: individuals with high stigma are more inclined to compare their own weight with “idealized bodies” displayed on social media, such as digitally retouched images, thereby reinforcing a fixed cognition of “I do not meet the standard,” which in turn devalues body image (35). Self-discrepancy theory elucidates the cognitive transformation stage: when individuals attribute the gap between their “actual self” (current weight) and their “ideal self” (internalized thinness standard) to “failure of self-discipline,” they develop persistent self-criticism, which accelerates the deterioration of body image (41). For example, individuals with high stigma often become trapped in a “binge–diet” cycle, during which their negative evaluations of body weight generalize into broader appearance anxiety (83). This process is reflected in distorted cognitions, such as expanding localized concerns (my legs are too thick) into global self-evaluations (everything I wear looks bad) or interpreting specific imperfections (my arms are not well-toned) as inevitable social ridicule (wearing short sleeves in summer will definitely invite mockery) (64). Such cognitive distortions exemplify overgeneralization, in which localized body features are forcibly linked to overall self-worth, thereby intensifying appearance anxiety (84).

### 4.4 Sample characteristics and comparison with previous studies

In comparison with previous studies, the sample in this study differs in several key aspects. First, our study focused specifically on female university students from six provinces of central and southern China. However, it is important to acknowledge that the sample’s homogeneity, consisting exclusively of female students from comprehensive universities in central and southern China, creates a narrow demographic scope, which may limit the generalizability of our findings to broader populations (85). Second, our sample was more inclusive in terms of academic year, including freshmen, sophomores, juniors, and seniors, whereas other studies often focused on one or two specific academic years. This broader inclusion may provide a more holistic view of how weight self-stigma and appearance anxiety develop across different stages of university education. Third, this study captured a larger sample size (n = 1,368), which contributes to the robustness and representativeness of the findings compared to previous studies with smaller samples (86). Thus, we acknowledge that future research should aim to include a more diverse sample, including students from various regions and educational backgrounds, to improve the external validity and applicability of the findings (87).

Furthermore, previous studies have demonstrated the significant impact of socioeconomic factors, such as family income and parental education, on body image and weight-related stigma (88–90). The absence of these factors in our study limits our ability to explore how weight self-stigma might vary across different socioeconomic strata (91, 92). Furthermore, BMI has consistently been found to be a critical variable in understanding weight-related stigma and appearance anxiety (61, 93, 94). The lack of BMI measurements in our study represents a key limitation. As BMI is directly related to self-perception of body weight, its absence prevents us from determining whether participants’ reported self-stigma accurately reflects their actual weight status.

### 4.5 Cultural context and theoretical considerations

This study was conducted within a specific East Asian cultural context, but it is important to recognize the cultural diversity both within this region and beyond (95). While our findings align with the widely documented “thin ideal” prevalent in East Asian beauty standards, they are far from monolithic across Eastern societies (96). For example, Japanese aesthetics have historically emphasized kawaii (cuteness) and youthful features (97), while Korean beauty standards often prioritize a small face and pale skin as markers of refinement (98). In Southeast Asia, beauty ideals may include more curvaceous body types or lighter skin tones, shaped by both local and global media influences (99). Within China itself, rapid economic development, urbanization, and pervasive exposure to Western and global media have given rise to a hybrid aesthetic landscape (100). Traditional Chinese values such as harmony with nature, moderation, and inner health (yangsheng) may coexist with, and at times conflict with, the modern pressures of market-driven appearance ideals (101–103). This interplay between traditional and contemporary values plays a significant role in how weight self-stigma is internalized and expressed (104).

Furthermore, the theoretical frameworks employed in this study—self-objectification theory (27) and social comparison theory (35)—were originally developed in Western, individualistic contexts, which calls for a critical evaluation of their applicability in collectivist East Asian societies (105). In cultures where the self is often defined in relation to others and social harmony is prioritized, weight stigma is experienced not only as a personal failure but also as a source of social disharmony or a failure to meet family expectations (106). This collectivist dimension may amplify the fear of enacted stigma, as negative evaluations are seen as reflecting poorly on one’s social group (107). At the same time, collectivist ties may offer stronger social buffers against appearance anxiety, which warrants further exploration (108). Future research should aim to develop culturally sensitive models that incorporate indigenous concepts, such as the Chinese notion of “face” (mianzi) or relational interdependence, to more accurately capture the psychological and social dynamics of weight stigma and appearance anxiety in Eastern populations (109, 110).

### 4.6 Contributions and limitations

From a variable-centered perspective, this study examined the mediating mechanism of body image in the relationship between weight self-stigma and appearance anxiety among female university students. From a person-centered perspective, it further identified distinct latent categories of weight self-stigma and body image. The findings highlight the positive role of body image in alleviating appearance anxiety triggered by weight self-stigma. This not only verifies and extends the functional domain of body image but also deepens the understanding of the mechanisms through which weight self-stigma exerts its influence (28).

This study makes important contributions at both theoretical and practical levels.

Theoretical contributions: By integrating both variable-centered and person-centered perspectives, this study not only confirmed the mediating role of body image in the pathway from weight self-stigma to appearance anxiety but also, for the first time, identified three latent profiles of weight self-stigma and body image among Chinese female university students (*Low Stigma–High Body Image*, *Moderate Stigma–Moderate Body Image*, and *High Stigma–Low Body Image*). These heterogeneous distribution patterns deepen the understanding of the complex relationship between weight stigma internalization and body-related cognition. Furthermore, the findings expand the applicability of self-objectification theory and social comparison theory in the East Asian cultural context (27, 35), providing a theoretical basis for the development of culturally sensitive psychological intervention models (111).

Practical contributions: The findings provide targeted guidance for mental health education and intervention strategies among female university students. Based on the latent profile analysis, educators and counselors can identify subgroups with different levels of risk and implement stratified interventions. For students in the *High Stigma–Low Body Image* group, cognitive-behavioral therapy may be applied to correct distorted body schemas. For those in the *Moderate Stigma–Moderate Body Image* group, interventions may emphasize social support and the promotion of diverse aesthetic values. Students in the *Low Stigma–High Body Image* group can serve as positive models for peer reinforcement and guidance. Moreover, the results suggest that universities should strengthen media literacy education to help students resist the dominance of the “slim–fair–young” aesthetic ideal and instead foster healthier and more diverse perspectives on body image, thereby mitigating the development and exacerbation of appearance anxiety at its source.

### 4.7 Limitations and future direction

This study also has several limitations. First, as a cross-sectional design was adopted, the findings can only reflect associations among the variables rather than causal relationships. Future research should employ longitudinal designs to reveal the dynamic and complex relationships over time. Second, the study included body image as the only mediating variable. Important covariates—such as social media use intensity, physical activity, eating behaviors, and mental health status—were not included in the analyses, potentially introducing omitted variable bias. Given that the mechanisms through which weight self-stigma influences appearance anxiety are multifaceted, other mediating or moderating variables may also play important roles. Future studies should incorporate additional psychological and contextual factors, including social media use, physical activity, and mental health status, to build a more comprehensive model. Third, the sampling approach presents certain constraints. Although purposive sampling in this study covered multiple provinces, diverse types of universities, and both rural and urban student sources, it remains a form of non-probability sampling. Consequently, the results primarily reflect the situation of female students in comprehensive universities in central and southern China. While this investigation may provide valuable insights into this specific demographic, the findings may not be fully generalizable to other regions of China, such as northern, western, or northeastern areas, where cultural, socio-economic, and educational factors may differ. The absence of participants from these regions limits the cultural breadth of the conclusions. Thus, caution is needed when generalizing these findings to broader populations, including students from vocational colleges or universities in other regions of China or other countries. Future research should consider stratified random sampling or other probability-based methods to enhance the representativeness, generalizability, and external validity of the conclusions.

Furthermore, the absence of objective BMI measurements and socioeconomic data in this study represents a significant limitation. The omission of key variables, such as family income, parental education, and socioeconomic background, prevents a more nuanced understanding of how weight self-stigma might vary across different social strata. These socioeconomic factors are well-established correlates of body image perception and stigma experiences, and their inclusion would offer a more comprehensive view of how social factors interact with body image issues. Future research should incorporate these variables to better capture the social dynamics at play. The lack of objective BMI measurements further undermines the validity of the study’s central variables. Weight self-stigma is inherently linked to actual body weight, and the absence of BMI data means we cannot determine whether participants’ reported self-stigma accurately reflects their true weight status or if it is influenced by distorted self-perceptions. This issue also limits our ability to explore perceptual accuracy, whether participants perceive their bodies accurately or experience distortions typical of eating disorders. Moreover, BMI could function as a moderating or confounding variable, influencing how self-stigma translates into appearance anxiety. The absence of this key variable limits the study’s interpretive depth. Therefore, future research should include BMI measurements to assess the relationship between weight self-stigma, body weight, and appearance anxiety more effectively.

## 5 Conclusion

1. Weight self-stigma positively predicted appearance anxiety among female university students.
2. Body image played a mediating role in the relationship between weight self-stigma and appearance anxiety.
3. Female university students could be classified into three latent classes based on weight self-stigma and body image: Low Stigma–High Body Image, Moderate Stigma–Moderate Body Image, and High Stigma–Low Body Image.
4. Compared with the Low Stigma–High Body Image group, both the Moderate Stigma–Moderate Body Image group and the High Stigma–Low Body Image group significantly and positively predicted appearance anxiety.

## Data Availability

The original contributions presented in the study are included in the article/supplementary material, further inquiries can be directed to the corresponding authors.
